# Reduced Rate of Inpatient Hospital Admissions in 18 German University Hospitals During the COVID-19 Lockdown

**DOI:** 10.3389/fpubh.2020.594117

**Published:** 2021-01-13

**Authors:** Lorenz A. Kapsner, Marvin O. Kampf, Susanne A. Seuchter, Julian Gruendner, Christian Gulden, Sebastian Mate, Jonathan M. Mang, Christina Schüttler, Noemi Deppenwiese, Linda Krause, Daniela Zöller, Julien Balig, Timo Fuchs, Patrick Fischer, Christian Haverkamp, Martin Holderried, Gerhard Mayer, Holger Stenzhorn, Ana Stolnicu, Michael Storck, Holger Storf, Jochen Zohner, Oliver Kohlbacher, Adam Strzelczyk, Jürgen Schüttler, Till Acker, Martin Boeker, Udo X. Kaisers, Hans A. Kestler, Hans-Ulrich Prokosch

**Affiliations:** ^1^Medical Center for Information and Communication Technology, Universitätsklinikum Erlangen, Erlangen, Germany; ^2^Department of Radiology, Universitätsklinikum Erlangen, Friedrich-Alexander-Universität Erlangen-Nürnberg (FAU), Erlangen, Germany; ^3^Chair of Medical Informatics, Friedrich-Alexander-Universität Erlangen-Nürnberg (FAU), Erlangen, Germany; ^4^Institute of Medical Biometry and Epidemiology, University Medical Center Hamburg-Eppendorf, Hamburg, Germany; ^5^Institute of Medical Biometry and Statistics, Medical Faculty and Medical Center, University of Freiburg, Freiburg, Germany; ^6^Institute of Medical Systems Biology, Ulm University, Ulm, Germany; ^7^Department of Nuclear Medicine, University Hospital Regensburg, Regensburg, Germany; ^8^Institute of Medical Informatics, Faculty of Medicine, Justus-Liebig-University, Gießen, Germany; ^9^Institute of Digitalisation in Medicine, Medical Faculty and Medical Center, University of Freiburg, Freiburg, Germany; ^10^Department of Medical Development and Quality Management, University Hospital Tübingen, Tübingen, Germany; ^11^Saarland University Medical Center, Institute for Medical Biometry, Epidemiology and Medical Informatics, Homburg, Germany; ^12^Institute for Translational Bioinformatics, University Hospital Tübingen, Tübingen, Germany; ^13^Institute of Medical Informatics, University of Münster, Münster, Germany; ^14^Medical Informatics Group, Universitätsklinikum Frankfurt, Frankfurt, Germany; ^15^Applied Bioinformatics, Department of Computer Science, University of Tübingen, Tübingen, Germany; ^16^Institute for Bioinformatics and Medical Informatics, University of Tübingen, Tübingen, Germany; ^17^Biomolecular Interactions, Max Planck Institute for Developmental Biology, Tübingen, Germany; ^18^Epilepsy Center Frankfurt Rhine-Main, Center of Neurology and Neurosurgery, Goethe University Frankfurt, Frankfurt, Germany; ^19^Department of Anesthesiology, University Hospital Erlangen, Erlangen, Germany; ^20^Institute of Neuropathology, Justus-Liebig-University, Gießen, Germany; ^21^Ulm University Medical Center, Ulm, Germany

**Keywords:** COVID-19, pandemic, healthcare systems, inpatient hospital admissions, Germany, medical informatics initiative, lockdown, university hospitals

## Abstract

The COVID-19 pandemic has caused strains on health systems worldwide disrupting routine hospital services for all non-COVID patients. Within this retrospective study, we analyzed inpatient hospital admissions across 18 German university hospitals during the 2020 lockdown period compared to 2018. Patients admitted to hospital between January 1 and May 31, 2020 and the corresponding periods in 2018 and 2019 were included in this study. Data derived from electronic health records were collected and analyzed using the data integration center infrastructure implemented in the university hospitals that are part of the four consortia funded by the German Medical Informatics Initiative. Admissions were grouped and counted by ICD 10 chapters and specific reasons for treatment at each site. Pooled aggregated data were centrally analyzed with descriptive statistics to compare absolute and relative differences between time periods of different years. The results illustrate how care process adoptions depended on the COVID-19 epidemiological situation and the criticality of the disease. Overall inpatient hospital admissions decreased by 35% in weeks 1 to 4 and by 30.3% in weeks 5 to 8 after the lockdown announcement compared to 2018. Even hospital admissions for critical care conditions such as malignant cancer treatments were reduced. We also noted a high reduction of emergency admissions such as myocardial infarction (38.7%), whereas the reduction in stroke admissions was smaller (19.6%). In contrast, we observed a considerable reduction in admissions for non-critical clinical situations, such as hysterectomies for benign tumors (78.8%) and hip replacements due to arthrosis (82.4%). In summary, our study shows that the university hospital admission rates in Germany were substantially reduced following the national COVID-19 lockdown. These included critical care or emergency conditions in which deferral is expected to impair clinical outcomes. Future studies are needed to delineate how appropriate medical care of critically ill patients can be maintained during a pandemic.

## Introduction

The COVID-19 pandemic has caused extreme strains on health systems, public health infrastructure, and economies of many countries. It has significantly impacted the German healthcare system on several levels. On March 16, 2020, the German Government announced first lockdown restrictions (1). For the healthcare system deferral of elective procedures was recommended to preserve hospital resources, especially intensive care beds, for COVID-19 patients (2). This lockdown disrupted routine hospital services for all non-COVID patients, for whom required treatments for non-urgent conditions were canceled or significantly postponed. The consequences for the quality of life and clinical outcomes of non-COVID-19 patients affected worldwide by COVID-19 lockdown regulations are not known. Moreover, a deterioration of the economic situation of the hospitals is to be expected as the DRG-based (diagnosis related groups) revenues will considerably decrease. As a result, the German Hospital Association (“Deutsche Krankenhausgesellschaft”) has already demanded to define a lump-sum budget for all German hospitals for the months April to December 2020 (3). It was estimated that more than 28 Mio. Surgical procedures have been canceled or postponed worldwide because of COVID-19 (4). In an early German correspondence, Kuhlen et al. (5) reported a 42.7% decrease in inpatient admissions during a 5-week period starting in mid March 2020 based on an analysis of 310 German hospitals that are part of the so-called “Initiative Qualitätsmedizin” (IQM). The highest decrease observed in their report was for knee endoprosthesis with 83.5% (5).

Importantly, surveys suggest that even patients with life-threatening conditions may have avoided hospital admission, possibly due to fear of SARS-CoV2 exposure. Thus, anecdotal observations (6) and reports especially focusing on cardiac and neurological procedures already note decreased patient numbers, for example, in the U.S. and Austria: Kansagara et al. describe a 39% decrease in the use of stroke imaging based on an analysis of the respective numbers in a commercial neuroimaging database across 856 hospitals in the U.S. (7), whereas Metzler et al. have conducted a nationwide retrospective survey and reported a similar major decline (39.4%) in hospital admissions/treatment during March 2020 for all subtypes of acute coronary syndrome with the beginning of the COVID-19 outbreak in Austria (8). In summary, the potential tangible effects of the COVID-19 pandemic on medical care for conditions other than COVID-19 have been difficult to quantify and, consequently, more comprehensive analyses are required (9). Thus, one of our research questions was to investigate if the reductions in inpatient hospital admissions for critical care conditions as reported in the international literature could be confirmed in our cohort.

Within the German Medical Informatics Initiative (MII), all German university hospitals started to establish data integration centers (DIC) in 2018 and 2019 for the purpose of managing, computing, and sharing data extracted from electronic health records (EHRs) (10). The participating hospitals are organized in four consortia (DIFUTURE, HiGHmed, MIRACUM and SMITH), all working on different approaches for data sharing (11–14).

The fundamental concept for all consortia and for cross-consortial cooperation, however, is a federated approach, that is, the data remain locally within each university hospitals' data repository and the analysis algorithms are distributed to the sites for joint analysis projects. At an early stage, this approach was already used to analyze regional differences in thrombectomy rates in stroke patients across the 10 MIRACUM university hospitals (15). In 2018/2019, the capabilities for data sharing across the sites of all MII consortia were illustrated by a demonstrator study, which focused on the analysis of comorbidity and rare diseases (16).

In response to the COVID-19 pandemic, the four consortia have rapidly assembled their joint expertise in data sharing infrastructures and established a concept for the National University Medicine (NUM) COVID-19 data and technology platform. The concept comprises a comprehensive set of decentralized as well as central components and will be published in the future. Here, we present first results of a preparatory groundwork, which was initiated by the MIRACUM consortium around its data extraction, transformation and loading (ETL) architecture based on a FHIR® gateway and the Informatics for Integrating Biology & the Bedside (i2b2) data repositories (17–19). From May to July 2020, the infrastructure was made available to all German university hospitals. Twenty of them implemented it in their DIC, but only eighteen of those obtained Institutional Review Board (IRB) as well as data Use and Access Committee (UAC) approval for joint analysis in this initial project before end of July 2020.

The objective of this study is to describe the change of care in the German COVID-19 lockdown phase by comparing the counts of inpatient hospital admissions and admissions related to specific clinical situations during the first 9 weeks (i.e., March 16 to May 17, 2020) of the German COVID-19 lockdown phase with the counts of corresponding timeframes in 2018 and 2019.

## Methods

This comparative retrospective study relates the number of events (as indicated below) during the lockdown period to the number of events in corresponding periods in 2018 and 2019 based on the analysis of a claims dataset across 18 German university hospitals. We have built on infrastructures and regulatory frameworks established at the participating university hospitals in the first 2 years of MII funding (2018/2019). All sites involved were in the process of setting up local DIC, extracting data from a variety of local data sources, harmonizing such data and preparing them locally for applying joint observational research analyses. Even though the four consortia have originally designed different architectural approaches for their respective DIC and for sharing data across their consortium sites, the national MII steering committee has initiated several working groups with the goal of encouraging and maintaining inter-consortial interoperability, consent, data sharing and communication. During the past years, these working groups developed, for example, a template form for acquiring a broad patient consent for reusing clinical data for research, common process definitions, regulations and governance structures for data sharing, as well as a joint core dataset specification, based on the international FHIR® standard. Most of the sites had already established data UACs, while some used this project to initiate their implementation.

### Inclusion Criteria

All patients fulfilling the following criteria were included in the study:

- Inpatient hospital admission between January 1 and May 31 of the years 2018, 2019, and 2020 respectively.
- All cases had to be complete, that is, a discharge date had to be present at the time of data retrieval.

Cases with a missing discharge date were excluded from the subsequent analysis.

### Outcomes

Main outcomes of the study are the number of *inpatient hospital admissions* and the number of admissions in derived groups as described below for the lockdown period and the corresponding periods in 2018 and 2019 at a temporal granularity of one calendar week (“cw”). Changes between periods are represented as absolute and relative differences.

Appropriate cases were grouped by ICD chapters (according to ICD-10-GM chapters) and reasons for treatment, defined by combining diagnosis codes and procedure codes [as described by Günster et al. (20)].

The following data elements were queried from each participating site's research data repository for eligible inpatient encounters:

- Principal diagnosis (primary codes based on the tenth revision of the International Statistical Classification of Diseases and Related Health Problems, German Modification, ICD-10-GM; www.dimdi.de).
- Related procedures [available as “Operationen- und Prozedurenschlüssel” (OPS)]
- Begin of inpatient stay (granularity of calendar days)
- End of inpatient stay (granularity of calendar days)
- Pseudonymized patient identifier (ID)
- Pseudonymized encounter ID.

### Data Acquisition and Data Governance

A dockerized (21) infrastructure environment, consisting of prepackaged ETL-processes (including data transformation and data pseudonymization), a FHIR-based gateway and an i2b2 research database (17, 18), was provided by the DIC team of the University Hospital Erlangen (UHE) to the participating sites of the German MII (Table 1). This infrastructure supported a quick start to load data items of the basic modules from the MII core dataset (i.e., demographics, encounter information, diagnosis, procedures, laboratory analysis and medication) into a common data model (defined by the i2b2 ontology). For this first analysis, specific ETL processes were developed to accept the German reimbursement claims dataset for inpatient care as input format [data formatted according to the data dictionary provided by the “InEK—Institut für das Entgeltsystem im Krankenhaus” (22)]. The present query was developed and validated at UHE, and then distributed to the participating sites as a dockerized shiny (23) web application, implemented in R (24), that integrated with the abovementioned infrastructure environment. By agreeing on a common data model, the environment distributed to all participating sites provided the means to perform the same SQL statements and analysis scripts across all partnering hospitals and to deliver standardized aggregated data back to the UHE DIC team. This analysis approach was based on concepts that have already been applied for earlier distributed analyses pursued within the federated MIRACUM DIC infrastructure (15, 25). Participating sites had to retrieve the respective docker container and subsequently prepare their local data to be loaded into the infrastructure environment. The dockerized query was then executed against the local i2b2 database, loading the data *via* SQL into an R session to further transform and aggregate the data and provide the results via the local web user interface. Finally, after local review and approval according to the local site's data governance guidelines, these aggregated results were uploaded to a secure platform at the UHE DIC by each participating site.

**Table 1:** Basic Characteristics of the participating 18 University Hospitals.

**Hospital**	**MII consortium**	**Inpatient hospital admissions 2018**	**Number of hospital beds**
University Hospital Tuebingen	DIFUTURE	74,091	1,585
Saarland University Medical Center	DIFUTURE	54,703	1,445
University Medical Center Ulm	DIFUTURE	49,890	1,274
University Hospital Regensburg	DIFUTURE	35,525	839
Hannover Medical School	HiGHmed	62,748	1,520
University Hospital Muenster	HiGHmed	57,026	1,448
University Hospital Freiburg	MIRACUM	71,469	1,616
University Hospital Erlangen	MIRACUM	65,200	1,394
University Hospital Magdeburg	MIRACUM	58,089	1,100
Carl Gustav Carus University Hospital Dresden	MIRACUM	57,101	1,410
University Hospital Giessen	MIRACUM	54,971	1,145
University Hospital Marburg	MIRACUM	53,289	1,080
University Hospital Frankfurt	MIRACUM	51,160	1,496
University Medicine Greifswald	MIRACUM	35,847	831
Jena University Hospital	SMITH	59,840	1,392
University of Leipzig Medical Center	SMITH	56,591	1,451
University Hospital Aachen	SMITH	49,233	1,502
University Hospital Halle	SMITH	40,799	982

This retrospective federated analysis was reviewed and approved by the ethics committee of the Erlangen-Nürnberg University (259_20 Bc). An informed consent was waived due to the retrospective design of this analysis and the use of de-identified data. All participating sites subsequently obtained approval for the proposed analysis by their local ethics committees as well as UACs.

### Data Transformation and Statistical Analysis

The aggregated data of all sites was collected and consolidated at UHE, where all subsequent statistical analyses were conducted with R version 4.0.0 (24). The data was only analyzed in a descriptive manner and is based on summary statistics. Comparisons of absolute counts between time periods of different years are reported as relative differences (in percentages). Graphics were created in R with ggplot2 (26) and ggpubr (27). We here report the admission numbers of several clinical situations in comparison of 2018 and 2020. As a *reference* for the variability in admissions numbers between non-COVID years, we further present the differences between 2018 and 2019.

In Germany, first lockdown restrictions were announced on March 16, 2020 in cw 12 (1). In this publication, we refer to this point in time as the “lockdown-announcement.”

In non-COVID-19 years, a decrease in inpatient hospital admissions can be observed in connection with holiday periods. As the timing of Easter holidays fluctuates between March and April, the corresponding cw of 2018 and 2019 were relatively adjusted to align with Easter holidays of 2020, analogous as described by Günster et al. (20), for a more exact comparison of the weeks post lockdown-announcement with the previous years (“adjusted weeks”). More precisely, the cw ending with Easter Sunday were aligned for the years 2018, 2019, and 2020, that is, cw 15 in 2020 corresponds to cw 13 in 2018 and cw 16 in 2019.

Due to the focus of this analysis and for the sake of simplicity, we assume the week of the lockdown-announcement (cw 12) being the adjusted week 0, with previous weeks numbered with negative (adjusted week −1, −2 etc.) and subsequent weeks with positive numbers (adjusted week 1, 2 etc.) relative to adjusted week 0.

In order to investigate the implications of the lockdown regulations, two periods of time were examined more in detail with a first 4-week phase in which stricter restrictions applied and a second 4-week phase in which these restrictions were partially relaxed again:

- Weeks 1 to 4 after lockdown-announcement, which are referred to as “adjusted weeks 0 to 3” (i.e., cw 10–13 in 2018, cw 13–16 in 2019, and cw 12–15 in 2020).
- Weeks 5 to 8 after lockdown-announcement, which are referred to as “adjusted weeks 4 to 7” (i.e., cw 14–17 in 2018, cw 17–20 in 2019, and cw 16–19 in 2020).

## Results

The results presented below were derived from 18 German university hospitals (with a total of about one Mio. inpatient hospital admissions per year), with four hospitals from the DIFUTURE, two from the HiGHmed, eight from the MIRACUM, and four from the SMITH consortium. Inpatient hospital admissions of the year 2018 of the 18 participating university hospitals are summarized in Table 1.

Within our dataset, an admission reduction from 2018 to 2020 is present for adjusted weeks 0 to 3 with a median decrease of 1,524 admissions (IQR: 743.5, Table 2), which represents a relative change of −15.4 to −43.9% per hospital (data not shown). Within the 5 months (January to May), the number of inpatient hospital admissions across all participating sites only differed slightly in 2018 and 2019 (i.e., 449,154 vs. 443,685, which represents a decrease of 1.2%), whereas the number of inpatient admissions in 2020 were reduced to 383,734, which represents a decrease of 14.6% compared to 2018 (Table 3).

**Table 2:** Change in overall hospital admissions.

**Time period**	**Comparison**	**Minimum**	**Median (IQR)**	**Maximum**	**Total**
January 1 to May 31	2018 to 2019	−5,770	238.5 (1,180)	2,461	−5,469
	2018 to 2020	−10,439	−3,343 (2,483.25)	−585	−65,420
Adjusted weeks 0 to 3_*_	2018 to 2019	−720	179.5 (296)	715	2,268
	2018 to 2020	−3,040	−1,524 (743.5)	−735	−28,250
Adjusted weeks 4 to 7*	2018 to 2019	−1,593	−279 (415.25)	303	−6,728
	2018 to 2020	−2,922	−1,274 (702)	−704	−26,076

Change in overall hospital admissions from 2018 to 2019 and 2018 to 2020 across all 18 participating sites for three different time periods (the complete period of January 1 to May 31, the adjusted weeks 0 to 3 and the subsequent adjusted weeks 4 to 7). Negative numbers indicate a reduction, whereas positive numbers indicate an increase in the respective yearly comparison (* weeks in 2018 and 2019 have been adjusted for Easter holidays in Germany: adjusted weeks 0 to 3 correspond to calendar weeks (“cw”) 12 to 15 2020 (cw 10-13 in 2018, cw 13-16 in 2019); adjusted weeks 4 to 7 correspond to cw 16 to 19 2020 (cw 14–17 in 2020; cw 17–20 in 2019).

**Table 3:** Inpatient hospital admissions.

**Year**	**Time period**	**Minimum**	**Median (IQR)**	**Maximum**	**Total**
2018	January 1 to May 31	14,934	24,333.5 (8,672.25)	37,825	449,154
	Adjusted weeks 0 to 3*	2,727	4,410.5 (1,420.25)	6,931	80,606
	Adjusted weeks 4 to 7*	2,879	4,668.5 (1,707)	7,174	85,953
2019	January 1 to May 31	15,655	24,330.5 (8,499)	33,954	443,685
	Adjusted weeks 0 to 3*	2,921	4,543 (1,579.75)	6,481	82,874
	Adjusted weeks 4 to 7*	2,838	4,415.5 (1,420.75)	5,943	79,225
2020	January 1 to May 31	13,064	20,889.5 (7,377)	28,856	383,734
	Adjusted weeks 0 to 3*	1,707	2,844.5 (896)	4,122	52,356
	Adjusted weeks 4 to 7*	1,915	3,424 (1,434.25)	4,521	59,877

Summary of the admissions from January 1 until May 31 across all 18 participating sites stratified by year (*weeks in 2018 and 2019 have been adjusted for Easter holidays in Germany: adjusted weeks 0 to 3 correspond to calendar weeks (“cw”) 12 to 15 2020 (cw 10–13 in 2018, cw 13–16 in 2019); adjusted weeks 4 to 7 correspond to cw 16 to 19 2020 (cw 14–17 in 2020; cw 17–20 in 2019).

In adjusted weeks 0 to 3, overall inpatient hospital admissions were 52,356 in 2020 compared to 80,606 in 2018, which corresponds to a decrease of 35%, whereas from 2018 to 2019, an increase of 2.8% (80,606 to 82,874) could be observed. In adjusted weeks 4 to 7, overall inpatient hospital admissions were 59,877 in 2020 compared to 85,953 in 2018, which corresponds to a decrease of 30.3%, while from 2018 to 2019, a decrease of 7.8% (85,953 to 79,225) could be observed across all 18 hospitals.

**Figure 1:**
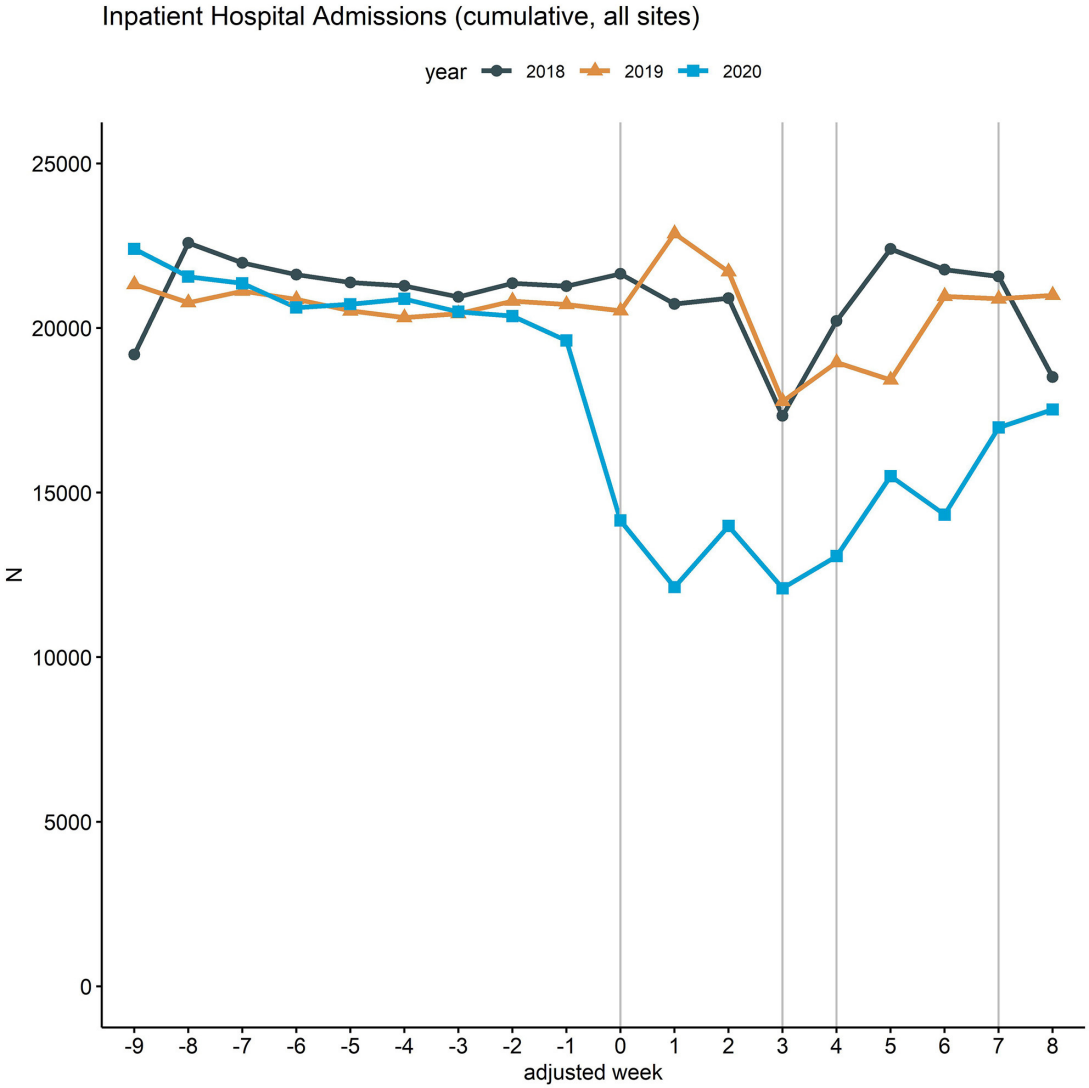
Inpatient hospital admissions in 2018, 2019, and 2020. Line chart of the cumulative hospital admissions per week across the 18 participating university hospitals. The curves are aligned for Easter holidays as outlined above. Vertical lines (gray) mark adjusted weeks 0, 3, 4 and 7, respectively.

Figure 1, which shows a line chart of the inpatient hospital admissions across all 18 hospitals from January 13 (adjusted week −9) to May 17, 2020 (adjusted week 8), illustrates these findings: A negative trend for inpatient hospital admissions can already be observed in 2020 for adjusted weeks −9 to −2 with a further decline after the complete lockdown in Italy (adjusted week −1), which coincides with the first COVID-19 related death in Germany. This decline is followed by a steep decline in the adjusted week 0, directly after the lockdown-announcement in Germany. In adjusted weeks 1 to 3 the admission numbers remain at a low level, whereas an increase can be observed in the subsequent 4 weeks (adjusted weeks 4 to 7).

Table 4 presents the number of inpatient hospital admissions grouped by the ICD-10 disease chapters I to IX in more detail: for three different time periods (the complete period of January 1, to May 31, the adjusted weeks 0 to 3 and the subsequent adjusted weeks 4 to 7) relative changes in admission rates of 2019 and 2020 are compared with the corresponding periods of 2018. Notably, all comparisons between 2018 and 2020 revealed a reduction of inpatient hospital admissions. In contrast, within the 5 months period from 2018 to 2019, the values in our cohort varied between an increase of 4.4% (ICD chapter I) and a decrease of −6.5% (ICD chapter III). Within the 4 weeks after the lockdown-announcement (adjusted weeks 0 to 3), the highest decrease of inpatient admissions in 2020 was associated with ICD chapters VIII “Diseases of the ear and mastoid process” (61.9%), followed by chapter XII “Diseases of the skin and subcutaneous tissue” (57.50%) and chapter XIII “Diseases of the musculoskeletal system” (56.7%) in comparison with the adjusted period of 2018. In contrast, the largest decrease from 2018 to 2019 could be observed with 23.2% in ICD chapter X “Diseases of the respiratory system.” The lowest reduction in admissions in 2020 were related to pregnancies/childbirths (chapter XV: 16.1%) and conditions originating in the perinatal period (chapter XVI: 8.1%) as well as neoplasms (chapter II: 12.9%).

**Table 4:** Comparison of the Inpatient Hospital Admissions Grouped by ICD Chapters.

	**January 1 to May 31**			Adjusted weeks 0 to 3*			Adjusted weeks 4 to 7*		
**ICD-10 chapter**	**No. of inpatient hospital admissions 2018**	**Rel. changes 2018 to 2019**	**Rel. changes 2018 to 2020**	**No. of inpatient hospital admissions 2018**	**Rel. changes 2018 to 2019**	**Rel. changes 2018 to 2020**	**No. of inpatient hospital admissions 2018**	**Rel. changes 2018 to 2019**	**Rel. changes 2018 to 2020**
I certain infectious and parasitic diseases	10,352	4.41%	−21.07%	1,841	13.96%	−36.99%	1,950	2.41%	−35.33%
II Neoplasms	88,976	−3.19%	−9.82%	15,472	4.85%	−12.85%	17,458	−11.75%	−23.61%
III diseases of the blood, blood-forming organs	4,149	−6.51%	−21.67%	650	16.46%	−24.00%	863	−18.77%	−35.11%
IV endocrine, nutritional and metabolic diseases	10,305	0.02%	−16.66%	1,723	16.77%	−39.99%	2,000	−10.10%	−34.40%
V mental and behavioral disorders	17,246	−6.24%	−19.58%	2,934	−1.26%	−44.17%	3,327	−14.16%	−39.10%
VI diseases of the nervous system	22,042	0.49%	−17.27%	3,951	4.35%	−51.10%	4,245	−4.90%	−36.49%
VII diseases of the eye and adnexa	25,569	0.70%	−20.32%	4,554	4.85%	−54.17%	5,061	−8.56%	−46.87%
VIII diseases of the ear and mastoid process	9,020	3.07%	−22.68%	1,652	6.11%	−61.86%	1,762	−11.12%	−50.23%
IX diseases of the circulatory system	49,181	1.84%	−12.04%	9,068	3.20%	−37.36%	9,188	−3.71%	−24.31%
X diseases of the respiratory system	25,308	−3.67%	−24.84%	5,539	−23.20%	−50.84%	4,123	−8.10%	−51.08%
XI diseases of the digestive system	28,815	2.03%	−13.44%	5,102	7.96%	−37.73%	5,468	−2.82%	−27.65%
XII diseases of the skin and subcutaneous tissue	9,966	−1.16%	−26.38%	1,673	12.79%	−57.50%	2,016	−16.17%	−54.42%
XIII diseases of the musculoskeletal system	21,150	−0.06%	−18.23%	3,644	3.54%	−56.67%	4,048	−6.65%	−40.07%
XIV diseases of the genitourinary system	19,803	0.80%	−12.31%	3,623	5.13%	−41.18%	3,767	−5.73%	−28.86%
XV pregnancy, childbirth and the puerperium	19,570	−1.03%	−6.96%	3,626	−2.90%	−16.08%	3,600	1.11%	−9.33%
XVI certain cond. origin. in the perinatal period	5,694	0.90%	−5.06%	1,057	−3.41%	−8.14%	1,067	0.75%	−10.03%
XVII congenital malformations	7,328	1.92%	−18.11%	1,357	9.21%	−49.89%	1,478	−8.73%	−37.55%
XVIII symptoms, signs and abnormal findings	15,636	−2.30%	−17.16%	2,847	−1.69%	−40.57%	2,888	−8.59%	−29.29%
XIX injury, poisoning and external causes	37,540	0.10%	−10.82%	6,459	10.50%	−28.64%	7,367	−3.35%	−21.05%

Comparison of the inpatient hospital admissions across all 18 participating hospitals, grouped by ICD chapters for three different time periods (the complete period of January 1 to May 31, the adjusted weeks 0 to 3 and the subsequent adjusted weeks 4 to 7). Relative changes in admission rates of 2019 and 2020 are compared with the corresponding periods of 2018 as a baseline (* weeks in 2018 and 2019 have been adjusted for Easter holidays in Germany: adjusted weeks 0 to 3 correspond to calendar weeks (“cw”) 12 to 15 2020 (cw 10–13 in 2018, cw 13–16 in 2019); adjusted weeks 4 to 7 correspond to cw 16 to 19 2020 (cw 14–17 in 2020; cw 17–20 in 2019).

In order to analyze the adaption of routine care processes to the restricted resources associated with COVID-19 lockdown and legislative regulations, we compared a set of clinical situations where the deferral of elective procedures would be seen as uncritical or at least less critical (i.e., arthrosis related endoprosthesis surgery, surgeries of benign tumors) to situations with more critical events such as endoprosthesis surgery after hip fracture, surgeries due to malignant tumors, myocardial infarction or stroke. The latter should be treated without any delay during a COVID-19 lockdown. Specifically, we analyzed critical events for which high reductions in inpatient hospital admissions were reported in the international literature [e.g., Hoyer et al.: 38 and 46% reduction of stroke admissions at 2 of 4 sites of a multi-center study in Germany (28); Rodríguez-Leor et al.: 40% reduction of STEMI-setting related procedures across 73 sites in Spain (29)].

**Figure 2:**
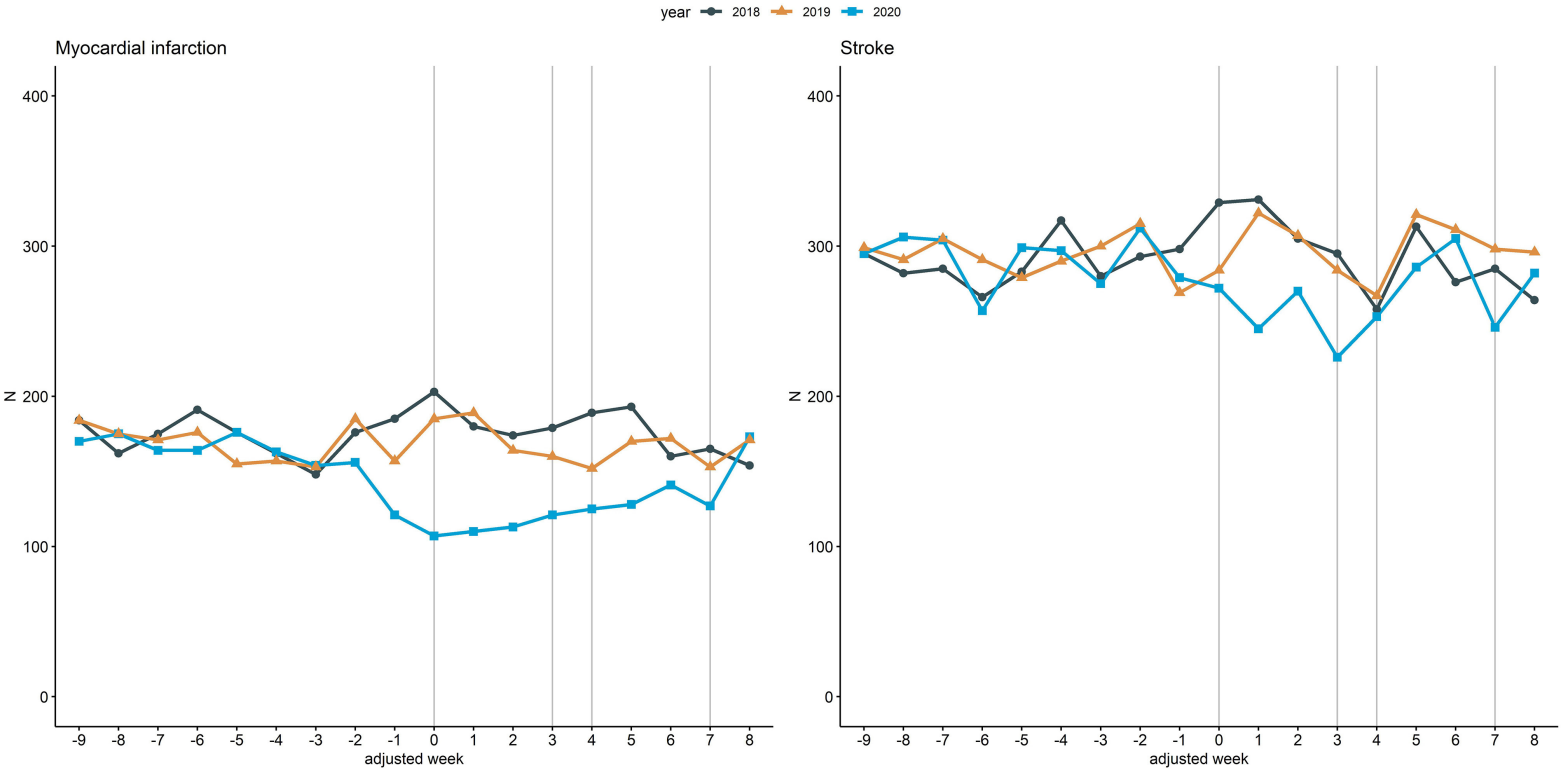
Inpatient hospital admissions related to myocardial infarction and stroke. Line chart of the cumulative hospital admissions per week related to myocardial infarction (left) and stroke (right) across the 18 participating university hospitals. The curves are aligned for Easter holidays as outlined above. Vertical lines (gray) mark adjusted weeks 0, 3, 4 and 7, respectively.

Figure 2 illustrates inpatient hospital admissions related to myocardial infarction and stroke. Within adjusted weeks 0 to 3, admissions related to myocardial infarction were reduced by 38.7% (736 to 451) from 2018 to 2020 and admissions related to stroke by 19.6% (1,260 to 1,013). In contrast, the respective reductions between 2018 and 2019 were 5.2% (736 vs. 698) and 5% (1,260 vs. 1,197), respectively.

**Figure 3:**
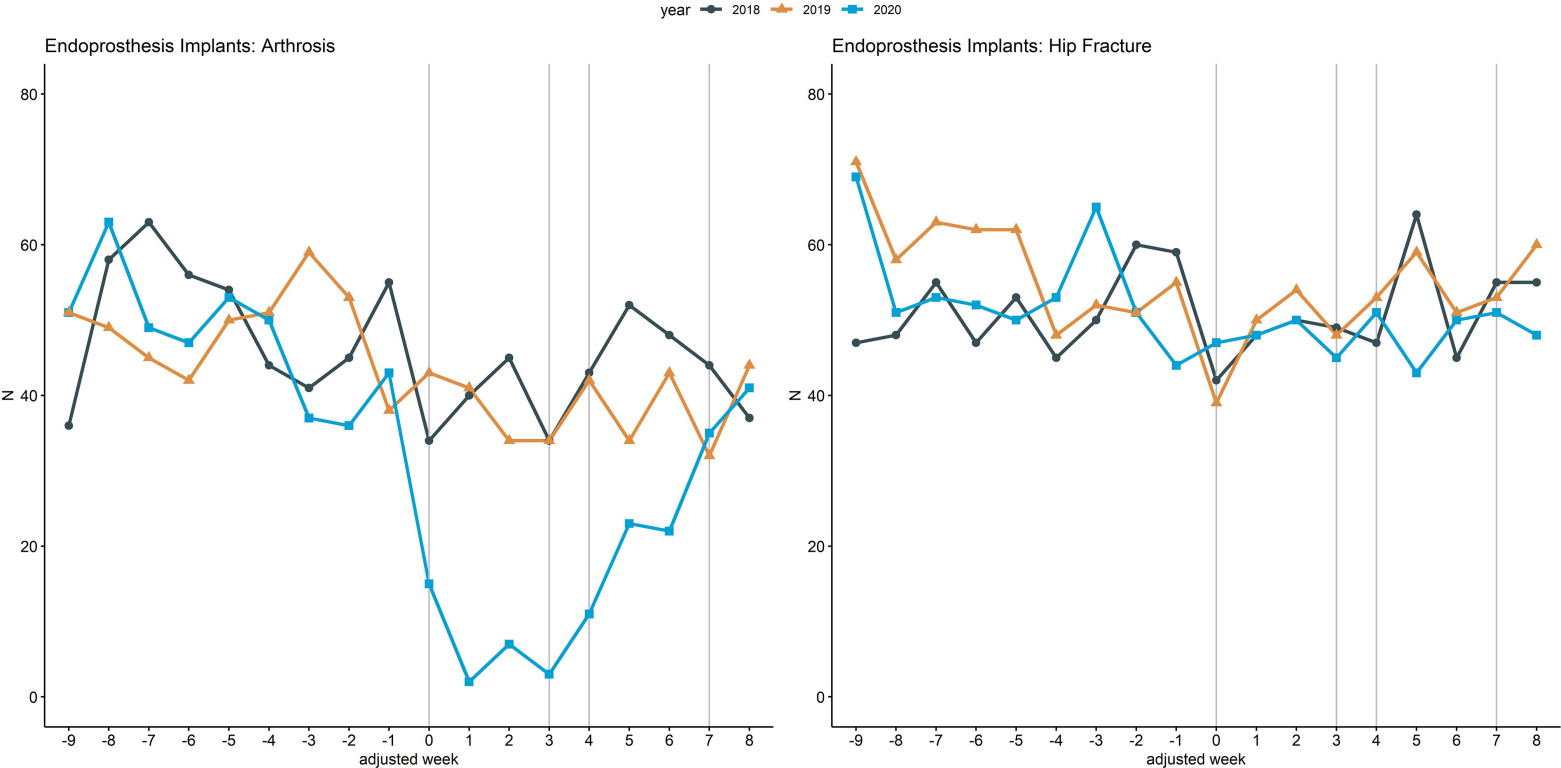
Inpatient hospital admissions related to endoprosthesis implants. Line chart of the cumulative hospital admissions per week related to endoprosthesis implants due to arthrosis (left) and due to hip fracture (right) across the 18 participating university hospitals. The curves are aligned for Easter holidays as outlined above. Vertical lines (gray) mark adjusted weeks 0, 3, 4, and 7, respectively.

Figure 3 illustrates inpatient hospital admissions related to endoprosthesis implants due to arthrosis and due to hip fracture, an example comparing a less critical with an emergency clinical situation. Within adjusted weeks 0 to 3, admissions related to endoprosthesis implants due to arthrosis were reduced by 82.4% (153 to 27) from 2018 to 2020, while admissions related to endoprosthesis implants due to hip fracture increased by 0.5% (189 to 190). In contrast, from 2018 to 2019 a reduction of 0.7% (153 vs. 152) could be observed for endoprosthesis implants due to arthrosis, whereas an increase of 1.1% (189 vs. 191) could be observed for admissions related to endoprosthesis implants due to hip fracture.

**Figure 4:**
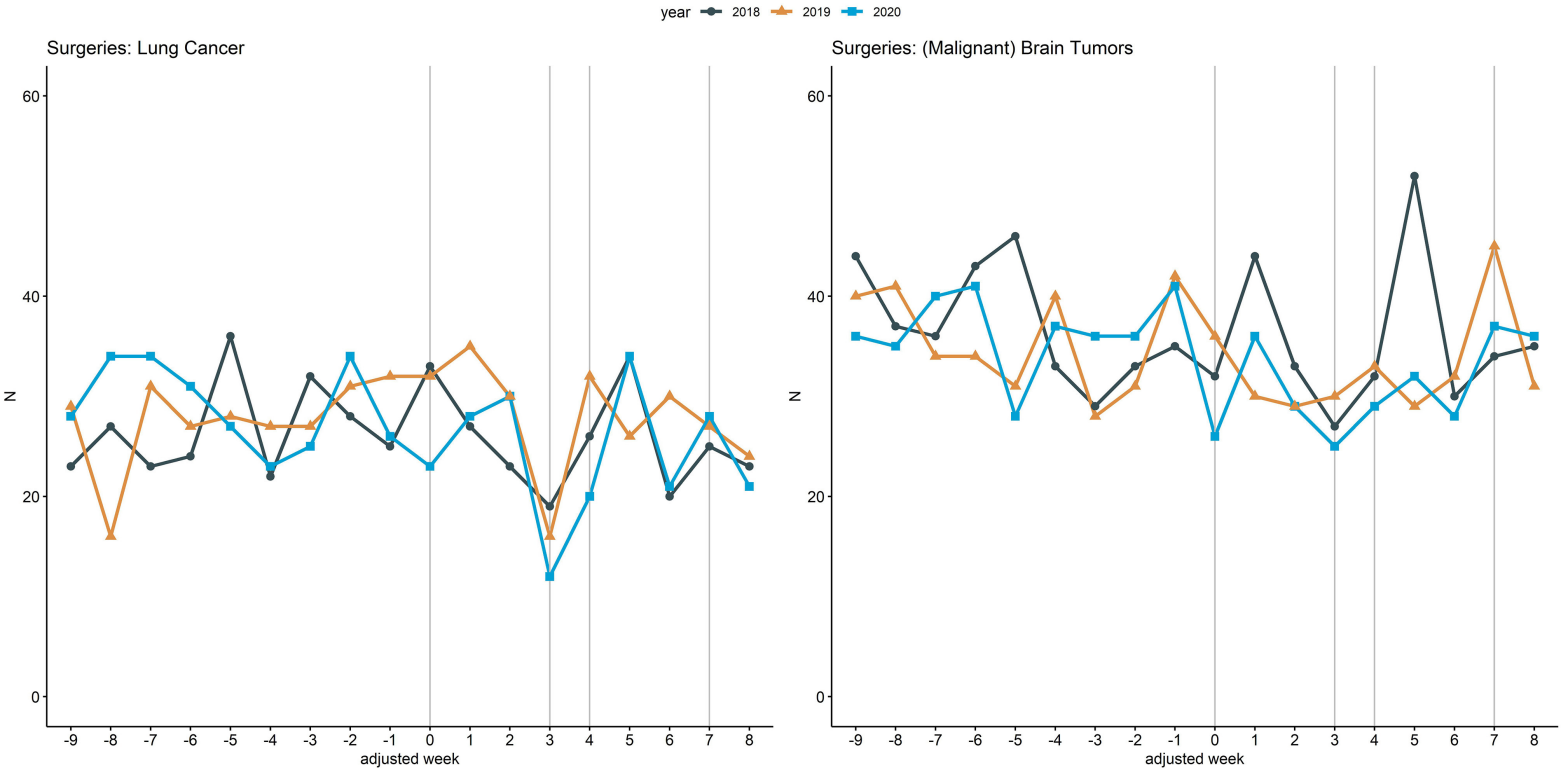
Inpatient hospital admissions related to lung cancer and brain tumor (malignant) related surgeries. Line chart of the cumulative hospital admissions per week related to lung cancer related surgeries (left) and due to (malignant) brain tumor related surgeries (right) across the 18 participating university hospitals. The curves are aligned for Easter holidays as outlined above. Vertical lines (gray) mark adjusted weeks 0, 3, 4, and 7, respectively.

Figure 4 illustrates inpatient hospital admissions related to lung cancer and (malignant) brain tumor surgeries, with the 2020 curve being similar to the curves of 2018 and 2019. Within adjusted weeks 0 to 3, admissions related to lung cancer surgeries were reduced by 8.8% (102 to 93) from 2018 to 2020 and admissions related to (malignant) brain tumor surgeries by 14.7% (136 to 116). In contrast, from 2018 to 2019 an increase of 10.8% (102 vs. 113) could be observed for lung cancer surgeries whereas a decrease of 8.1% (136 vs. 125) could be observed for (malignant) brain tumor related surgeries.

**Figure 5:**
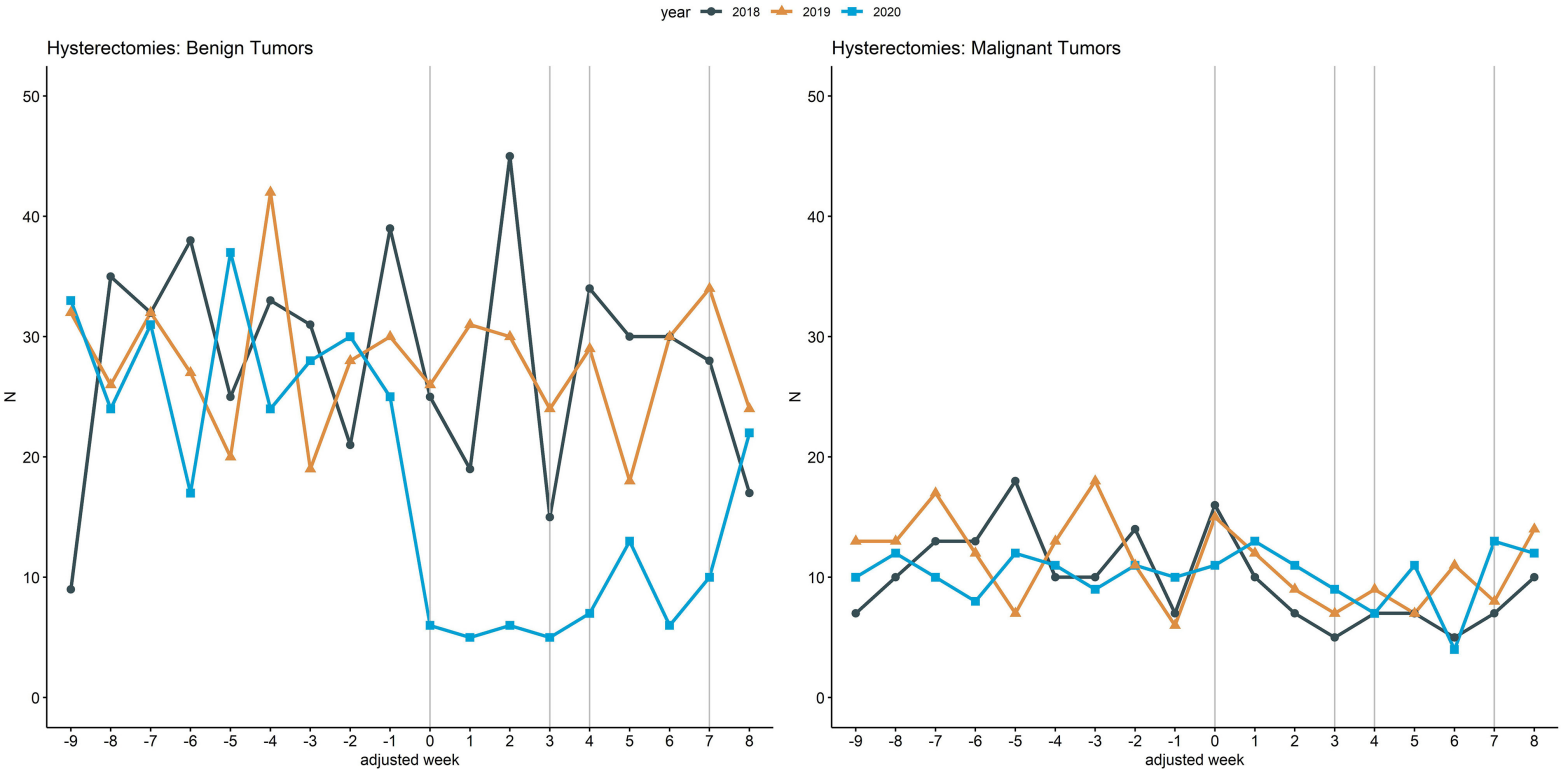
Inpatient hospital admissions related to hysterectomies. Line chart of the cumulative hospital admissions per week related to hysterectomies in case of benign tumors (left) and due to hysterectomies of malignant tumors (right) across the 18 participating university hospitals. The curves are aligned for Easter holidays as outlined above. Vertical lines (gray) mark adjusted weeks 0, 3, 4, and 7, respectively.

Figure 5 illustrates inpatient hospital admissions related to hysterectomies due to benign tumors and due to malignant tumors, a further example comparing a less critical with a more critical clinical situation. Within adjusted weeks 0 to 3, admissions related to hysterectomies due to benign tumors were reduced by 78.8% (104 to 22) from 2018 to 2020 and increased by 15.8% (38 to 44) for hysterectomies due to malignant tumors. In contrast, from 2018 to 2019 increases of 6.7% (104 vs. 111) and 13.2% (38 vs. 43) were observed for these clinical situations.

## Discussion

In this descriptive retrospective analysis, a decrease in overall inpatient hospital admissions of 35% was observed across 18 university hospitals in Germany in 2020 during the 4 weeks directly after the announcement of the lockdown due to COVID-19 compared to the adjusted period of 2018. In contrast to this, a slight increase of 2.8% could be observed from 2018 to 2019. The decrease from 2018 to 2020 is similar to the decrease of 39% reported by Günster et al. (20), who analyzed a cohort of members of the statutory sickness fund “AOK” and were treated in German hospitals between March 16 and April 5, 2020. Kuhlen et al., however, reported an even larger decrease of 42.7% in inpatient hospital admission between March 3 and April 19, 2020 in comparison with 2019 (5).

Thus, overall inpatient hospital admissions in our investigation showed a slightly lower decrease than those reported by Kuhlen et al. (5) and Günster et al. (20), which may have several reasons: First, total numbers of cases analyzed differ among the three studies. While our study cohort consisted of 52,356 inpatient hospital admissions in the 4 weeks after the lockdown-announcement, Günster et al. and Kuhlen et al. analyzed 240,774 cases and 294,622 cases, respectively. Second, the three studies analyzed slightly different time periods in 2020, which are, however, overlapping by the 3 weeks from March 16 to March April 5, 2020 (adjusted weeks 0 to 2). Furthermore, we have analyzed data exclusively from university hospitals. This could explain, why inpatient hospital admissions were slightly less reduced in our analysis. Lastly, it should be noted that, analogous to Günster et al. (20), we compared the numbers of 2020 with holiday adjusted weeks of 2018 and 2019 to control for the decline in inpatient hospital admissions associated with the Easter holidays in Germany, which was observed in our cohort.

Our results regarding inpatient hospital admissions due to myocardial infarctions (−38.7% from 2018 to 2020) are in accordance with those reported by Günster et al. (20) who found a 31% reduction within the “AOK” dataset in comparison with 2019. Both are similar to the 38% reduction reported by US cardiac catheterisation laboratories (30), but lower than the rates reported from Spain [i.e., a 40% reduction of STEMI-setting related procedures (29)], and Italy, where a reduction of 40–50% of acute coronary syndrome related admissions to an intensive cardiac care unit was observed in comparison with 2019 (31). This effect may have been caused by the more restrictive lockdown regulations in these two countries. However, the reduction in myocardial infarctions observed in our study was much higher than the 25% reduction found in a dedicated analysis of the German health insurance company DAK from April 2020 (32) and the 22% reduction for cardiac events in 2020 compared to the average of 2017 to 2019 reported from Ulm University Hospital (33).

While other researchers observed a reduction of up to 46% in admission rates for stroke patients (28), our analysis across the 18 German university hospitals shows a reduction of 19.6% from 2018 to 2020. This lesser reduction might be due to our analysis of acute ischemic or hemorrhagic stroke which are associated with neurological deficits and imaging findings of ischemia or hemorrhage, since we did not include patients with transient ischemic attack (TIA). Patients with TIA, who experience minor and transient neurological deficits, might have in particular deferred hospital admission during the COVID-19 lockdown. Our findings, however, are in accordance with those reported by Günster et al. who reported a reduction of 19% in stroke admissions compared to 2019 (20).

The careful prioritization of critically ill patients by the 18 German hospitals is illustrated by the differences in hospital admissions for endoprosthesis implants due to arthrosis and due to hip fracture. In the 4 weeks after the lockdown-announcement in Germany, admissions due to hip fractures were similar compared to the adjusted period in 2018 [< -1 % vs. 2020, −2 % reported by Günster et al. (20)] whereas admissions for endoprosthesis implants due to arthrosis were reduced by 82.4% [79% decrease reported by Günster et al. in comparison with 2019 (20)]. The high similarity of our results to those of Günster et al. (20) confirms their earlier results and illustrates that urgent care for hip fractures apparently was not reduced within the COVID-lockdown phase, in Germany, in contrast to other European countries (34, 35).

Even though the observed trend in our results concerning cancer care during the COVID-19 pandemic are in alignment with reports from the US, our decreases are less pronounced. London et al. reported clear trends suggesting a significant decline of care in the cancer cohorts explored in their recent analysis of 20 US healthcare institutions based on the TriNetX COVID and Cancer Research Network (between 39.1% reduction for lung cancer and 51.8% reduction for melanoma) (36). When analyzing the inpatient hospital admissions grouped by ICD chapters, we observed an overall reduction of only 12.9% in neoplasm-related admissions (ICD chapter II) during the first 4 weeks after lockdown-announcement compared to 2018. Our analyses regarding other cancer related inpatient hospital admissions show similar results with a decrease of 8.8% for lung cancer related surgeries and 14.7% for (malignant) brain tumor related surgeries in 2020 compared to 2018. Günster et al. report a reduction of 20% for admissions due to lung cancer related surgeries from 2019 to 2020, whereas the reduction in hospital admissions for (malignant) brain tumor related surgeries was only 2% (20). However, it has to be noted that the decrease from 2018 to 2020 in our study is based on a difference of only 9 admissions for lung cancer and 20 admissions for (malignant) brain tumors.

A further stringent example for the careful prioritization of critically ill patients by the 18 German hospitals are the inpatient hospital admissions related to hysterectomies due to benign tumors compared to the ones due to malignant tumors: hysterectomies due to benign tumors were strongly reduced by 78.8% but even moderately increased in 2020 by 15.8% if due to malignant tumors compared to 2018. Günster et al. reported a reduction of 66% for hysterectomies due to benign tumors and an increase of 23% for malignant tumors from 2019 to 2020 (20). While the trend of our results regarding hysterectomies is in alignment with the numbers observed by Günster et al., it has to be noted, that the reduction observed from 2018 to 2020 in our study is based on a difference of only six cases for malignant tumors. However, our analysis of admissions related to hysterectomies underlines that the university hospitals in our cohort adapted their care practices to the urgency of the clinical situation.

This study has a number of limitations: First, it is a retrospective analysis based on observational data collected in EHR. Second, the overall sample size for many of the here investigated clinical situations is small with a large variability between the 18 participating hospitals. Because of the large heterogeneity between German hospitals, for example, in number of beds, number of admissions and case mixes (also within those included in our study, compare Table 1) our data cannot be seen as a full representative picture for all German university hospitals. Therefore, we present only descriptive results and have resigned from statistical hypothesis testing. Furthermore, other co-variates and possible confounding variables have not been controlled, for example, the development of the demographic situation in Germany, the catchment area of the respective hospitals, different age and gender distributions, or different availability and distribution of specialist departments at the participating hospitals. One of the most important limitations is that data was drawn from sets of claims data having a standardized format used for quality assurance and for the calculation of the new versions of the German DRG system each year (see §21 KHEntgG, hospital renumeration law). The procedure and quality assurance measures for providing this dataset until the yearly deadline (March 31) are highly standardized. However, even though datasets with this data structure are regulated by law, the data set may differ slightly among hospitals, if it is generated during the year (e.g., some hospitals might exclude cases which have no discharge event yet from the observed period, others might include them). In order to ensure a standardized approach across all participating sites, we strictly adhered to the legal requirements to format the dataset and thus, for example, excluded cases without a discharge date from the analyses. Despite its limitations, the existing data set provides the best information currently available at German university hospitals for this study.

Those limitations constrain the conclusions we can currently draw. However, future studies could add more detail to our findings presented here. Furthermore, we believe that the sources of our data and the technologies, procedures and regulatory framework we have established for sharing them are sound, reproducible and scalable. The latter is the most important aspect for our project to implement the National University Medicine (NUM) COVID-19 data and technology platform which will now be extended to include all German university hospitals and to cover a broad range of clinical data based on the basic modules of the MII core dataset and its extension with the GECCO dataset [GECCO = German Corona Consensus (37)].

Although we observed large heterogeneity across the 18 participating sites, indicated by the large inter-quartile ranges (IQR) in Table 3, an admission reduction from 2018 to 2020 is present across all 18 participating university hospitals during the 4 weeks following the announcement of the lockdown (Table 2). When using such observational data for statistical modeling in the future, the heterogeneity between sites needs to be taken into account, which is particularly relevant for future analysis based on the NUM data and technology platform. Although we expect that some part of the heterogeneity could be explained by different case mixes, external site-specific characteristics, for example, number of incident COVID-19 cases in the site's catchment area, results will also depend on internal site-specific characteristics, for example, availability of resources. Thus, global and local effects as well as their interactions need to be differentiated carefully in future analyses.

Within the German Medical Informatics Initiative, all German university hospitals have started to establish DIC for the purpose of managing, computing, and sharing data extracted from EHRs (10). In response to the COVID-19 pandemic, the four consortia have rapidly assembled their joint expertise in data sharing infrastructures and established a concept for the National University Medicine (NUM) COVID-19 data and technology platform. Even though this analysis was based on a first preparatory groundwork and not all German university hospitals could yet participate, it nevertheless illustrates the potential of such a federated network, which will be extended further in the upcoming months.

As a result of this early effort, we report the results of our analysis of inpatient hospital admissions within the German COVID-19 lockdown phase compared to the corresponding periods in 2018 and 2019, and thus illustrate the change of care during the lockdown. Within this work we cannot only reproduce and complement other national and international studies with results from German university hospitals but can also show that the Medical Informatics Initiative's approach to distributed data analysis works for large-scale projects.

In summary, our study shows that the hospital admission rates in Germany were substantially reduced following the national COVID-19 lockdown. Notably, these reductions included critical clinical situations in which deferral is expected to severely impair quality of life and clinical outcomes. Future studies are needed to delineate how appropriate medical care of critically ill patients can be maintained during a pandemic in particular in the light of the anticipated “second wave” of COVID-19.

## Data Availability Statement

The raw data supporting the conclusions of this article will be made available by the authors, without undue reservation.

## Ethics Statement

This retrospective federated analysis was reviewed and approved by the ethics committee of the Friedrich-Alexander-University Erlangen-Nürnberg (FAU) (259_20 Bc). An informed consent was waived due to the retrospective design of this analysis and the use of de-identified data. All participating sites subsequently obtained approval for the proposed analysis by their local ethics committees as well as UACs.

## Author Contributions

H-UP and LAK: conceptualization. H-UP, LAK, and MK: project administration. LAK: conceived and designed the analysis. MK, JG, CG, SM, and ND: software architecture and development. JM, JB, TF, PF, GM, HSte, ASto, HSto, JZ, OK, and UK: data acquisition. LAK and LK: performed the analysis. LAK: visualization. SS, JM, and SM: validation. LAK and H-UP: writing – original draft. LAK, H-UP, MB, TA, JS, MK, SS, AStr, CS, SM, JG, LK, DZ, JB, JM, CG, ND, TF, PF, CH, MH, GM, HS, MS, HS, JZ, OK, UK, and HK: writing – review and editing. All authors contributed to the article and approved the submitted version.

## Conflict of Interest

The authors declare that the research was conducted in the absence of any commercial or financial relationships that could be construed as a potential conflict of interest.

## MII-Research Group

- Jonas Bienzeisler, Medical Faculty, Institute of Medical Informatics, Medical Faculty, RWTH Aachen University, Aachen, Germany.
- Stephanie Biergans, Institute for Translational Bioinformatics, University Hospital Tübingen, Tübingen, Germany.
- Philipp Bornfleth, Data Integration Center, Otto-von-Guericke-University, Magdeburg, Germany.
- Andreas Dürschmid, Data Integration Center, University of Leipzig Medical Center, Leipzig, Germany.
- Jonas Fortmann, Institute of Medical Informatics, Medical Faculty, RWTH Aachen University, Aachen, Germany.
- Stefan Franz, Peter L. Reichertz Institute for Medical Informatics of TU Braunschweig and Hannover Medical School, Hannover, Germany.
- Denis Gebele, Institute of Medical Biometry and Statistics, Medical Faculty and Medical Center, University of Freiburg, Freiburg, Germany.
- Matthias Gietzelt, Peter L. Reichertz Institute for Medical Informatics of TU Braunschweig and Hannover Medical School, Hannover, Germany.
- Silke Haferkamp, Data Integration Center, IT Department, University Hospital RWTH Aachen, Aachen, Germany.
- Sven Helfer, Institute for Medical Informatics and Biometry, Carl Gustav Carus Faculty of Medicine, Technische Universität Dresden, Dresden, Germany.
- Alexander Helhorn, Data Integration Center, Jena University Hospital, Jena, Germany.
- Dirk Hellwig, Department of Nuclear Medicine, University Hospital Regensburg, Regensburg, Germany.
- Anton Köstlbacher, IT Department, University Hospital Regensburg, Regensburg, Germany.
- Petar Horki, Institute of Medical Biometry and Statistics, Medical Faculty and Medical Center, University of Freiburg, Freiburg, Germany.
- Dennis Kadioglu, Medical Informatics Group, University Hospital Frankfurt, Frankfurt, Germany.
- Christian Kamann, Institute for Medical Informatics and Biometry, Carl Gustav Carus Faculty of Medicine, Technische Universität Dresden, Dresden, Germany.
- Detlef Kraska, Medical Center for Information and Communication Technology, Universitätsklinikum Erlangen, Erlangen, Germany.
- Thomas Litschko, Department of Epidemiology of Health Care and Community Health, Institute for Community Medicine, University Medicine Greifswald, Greifswald, Germany.
- Irina Lutz, Data Integration Center, IT Department, University Hospital RWTH Aachen, Aachen, Germany.
- Raphael Majeed, Institute of Medical Informatics, Medical Faculty, RWTH Aachen University, Aachen, Germany.
- Jan Maluche, Data Integration Center, Otto-von-Guericke-University, Magdeburg, Germany.
- Kurt Marquardt, University Hospital Giessen, Gießen, Germany.
- Daniel Medenwald, Department of Radiation Oncology, University Hospital Halle, Halle (Saale), Germany.
- Achim Michel-Backofen, University Hospital Giessen, Giessen, Germany.
- Peter Penndorf, Department of Epidemiology of Health Care and Community Health, Institute for Community Medicine, University Medicine Greifswald, Greifswald, Germany.
- Ariadna Perez Garriga, Institute of Medical Informatics, Medical Faculty, RWTH Aachen University, Aachen, Germany.
- Rainer Röhrig, Institute of Medical Informatics, Medical Faculty, RWTH Aachen University, Aachen, Germany.
- Christian Scheer, Department of Anesthesiology, University Hospital Greifswald, Greifswald, Germany.
- Sebastian Schindler, Data Integration Center, Otto-von-Guericke-University, Magdeburg, Germany.
- Henning Schneider, Institute of Medical Informatics, Faculty of Medicine, Justus-Liebig-University, Gießen, Germany.
- Christian Seidemann, Data Integration Center, Philipps-University of Marburg, Marburg, Germany.
- Abel Stolz, Data Integration Center, University of Leipzig Medical Center, Leipzig, Germany.
- Daniel Tiller, IT Department, Data Integration Center, University Hospital Halle, Halle (Saale), Germany.
- Hauke Tönnies, Institute of Hygiene, University Hospital Münster, Münster, Germany.
- Thomas Wendt, Data Integration Center, University of Leipzig Medical Center, Leipzig, Germany.
- Emilia Wisotzki, Data Integration Center, IT Department, University Hospital Halle, Halle (Saale), Germany.
- Nico Wöller, Department of Epidemiology of Health Care and Community Health, Institute for Community Medicine, University Medicine Greifswald, Greifswald, Germany.
